# PathCase-SB: integrating data sources and providing tools for systems biology research

**DOI:** 10.1186/1752-0509-6-67

**Published:** 2012-06-14

**Authors:** Sarp A Coskun, Xinjian Qi, Ali Cakmak, En Cheng, A Ercument Cicek, Lei Yang, Rishiraj Jadeja, Ranjan K Dash, Nicola Lai, Gultekin Ozsoyoglu, Zehra Meral Ozsoyoglu

**Affiliations:** 1 Electrical Engineering and Computer Science Department, Case Western Reserve University, Cleveland, USA; 2 Department of Physiology, Medical College of Wisconsin, Milwaukee, USA; 3 Department of Biomedical Engineering, Case Western Reserve University, Cleveland, USA; 4 Department of Pediatrics, Case Western Reserve University, Cleveland, USA

## Abstract

**Background:**

Integration of metabolic pathways resources and metabolic network models, and deploying new tools on the integrated platform can help perform more effective and more efficient systems biology research on understanding the regulation of metabolic networks. Therefore, the tasks of (a) integrating under a single database environment regulatory metabolic networks and existing models, and (b) building tools to help with modeling and analysis are desirable and intellectually challenging computational tasks.

**Results:**

PathCase Systems Biology (PathCase-SB) is built and released. This paper describes PathCase-SB user interfaces developed to date. The current PathCase-SB system provides a database-enabled framework and web-based computational tools towards facilitating the development of kinetic models for biological systems. PathCase-SB aims to integrate systems biology models data and metabolic network data of selected biological data sources on the web (currently, BioModels Database and KEGG, respectively), and to provide more powerful and/or new capabilities via the new web-based integrative framework.

**Conclusions:**

Each of the **c**urrent four PathCase-SB interfaces, namely, Browser, Visualization, Querying, and Simulation interfaces, have expanded and new capabilities as compared with the original data sources. PathCase-SB is already available on the web and being used by researchers across the globe.

## Background

Integrating selected data from multiple data sources with the goals of expanding the capabilities of original data sources, and allowing new tool-building opportunities is a common theme in many fields of computer science. PathCase Systems Biology (*PathCase-SB*) [1,2] is such a site, released on Aug. 2010, that brings together the data of (i) systems biology data sources, e.g., BioModels Database [3-5], and (ii) pathways data sources, e.g., KEGG [6-9], with the goal of providing additional capabilities and tools made possible due to the integration. By pathways, we refer to pathways of various different metabolism as defined by biochemists, and as found in biochemistry textbooks (such as [10]) and atlases (such as [11]). In this paper, we describe the current functionality (i.e., the currently available user interfaces) of PathCase-SB which provides a database-enabled integrative framework and tools towards effective and efficient systems biology model development and simulation for mechanistic understanding of the behavior of complex biological systems.

PathCase-SB is web-based and has multiple interfaces:

· Visualization Interface to visualize the networks of models and/or pathways in multiple ways,

· Browser Interface to browse models and pathways in multiple ways,

· Querying Interface to query models and pathways in different ways,

· Simulation Interface to comparatively simulate models, and

· Model Composition Interface to compose new models from existing models

PathCase-SB has an API for others to tap into, and to perhaps build additional applications, such as a mobile-tablet access to PathCase-SB database (see multiple PathCase iPad applications iPathCase-KEGG, iPathCase—RCMN, and iPathCase-SMDA [12]). PathCase-SB belongs to a family of PathCase applications [13-15], where each application is designed for different purposes and use.

PathCase-SB is not a model/pathways-data source. It does not provide systems biology model curation, which is already provided in an excellent manner by other data sources—namely, BioModels Database for SBML-based models [16] and CellML model repository [17,18] for CellML-based models. In addition, PathCase-SB is not a primary data source for pathways. Instead, PathCase-SB relies on (a) freely available (and curated-only) models in BioModels Database and CellML model repository to populate its model database, and (b) pathways from KEGG pathways data source [6], obtained via a license, to populate its pathways database. See Cakmak et al., 2011 [2] for the details of PathCase-SB software architecture and database. The goal of PathCase-SB is to provide a semantics-based environment for systems biology researchers to

1. Explore (browse) and query both models, their networks, and pathways--perhaps with the goal of, say, selecting some systems biology models or their modeled reactions (e.g., ad hoc exploratory search capabilities)

2. Visualize, possibly comparatively, both pathways and/or networks of models--perhaps with the goal of, say, visually inspecting the modeled species, reactions, and their occurrences in their corresponding KEGG pathways (e.g., ad hoc exploratory visualization capabilities)

3. Comparatively simulate, possibly with users’ own experimental data, few selected models and/or users’ own models uploaded into PathCase-SB (e.g., comparative simulation capabilities with users’ own experimental data), and

4. Compose new models by using the existing models in the PathCase-SB database

A key characteristic of PathCase-SB is, via multiple interfaces, to enable systems biology researchers to synergistically use bioinformatics tools such as model and pathway database repositories, pathway visualization, SBML simulators, provenance data, and metabolic system queries in the study of complex biological systems.

PathCase-SB is continually being developed and improved. The current version of PathCase-SB integrates BioModels Database and KEGG data, and has the first-release functionalities for items 1–4. For the next release, planned additions include (i) incorporating models in CellML model repository and Reactome [19] pathways into PathCase-SB, and (iii) rewriting various interfaces for increased response time.

**Figure 1:**
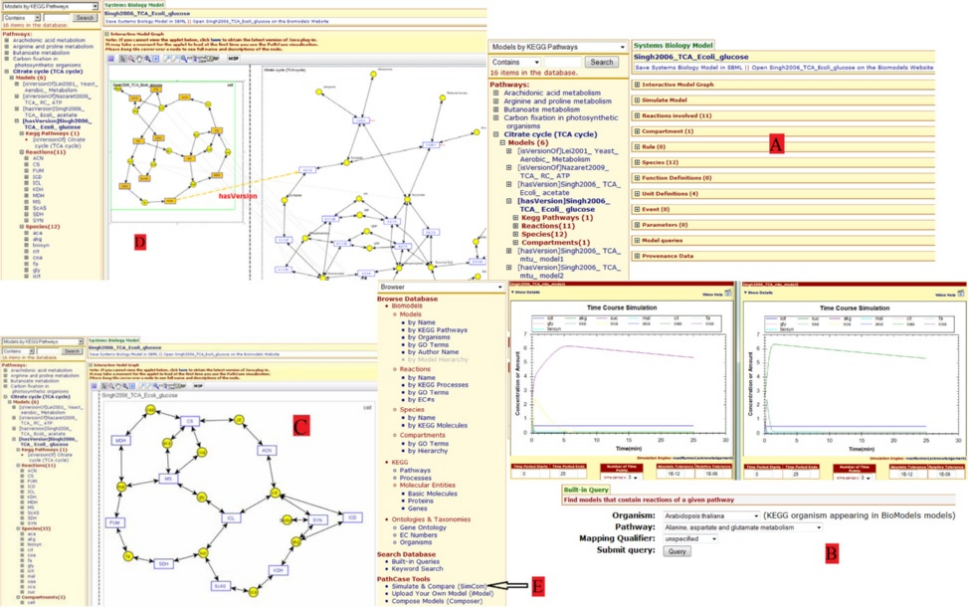
Example 1.1.

**Figure 2:**
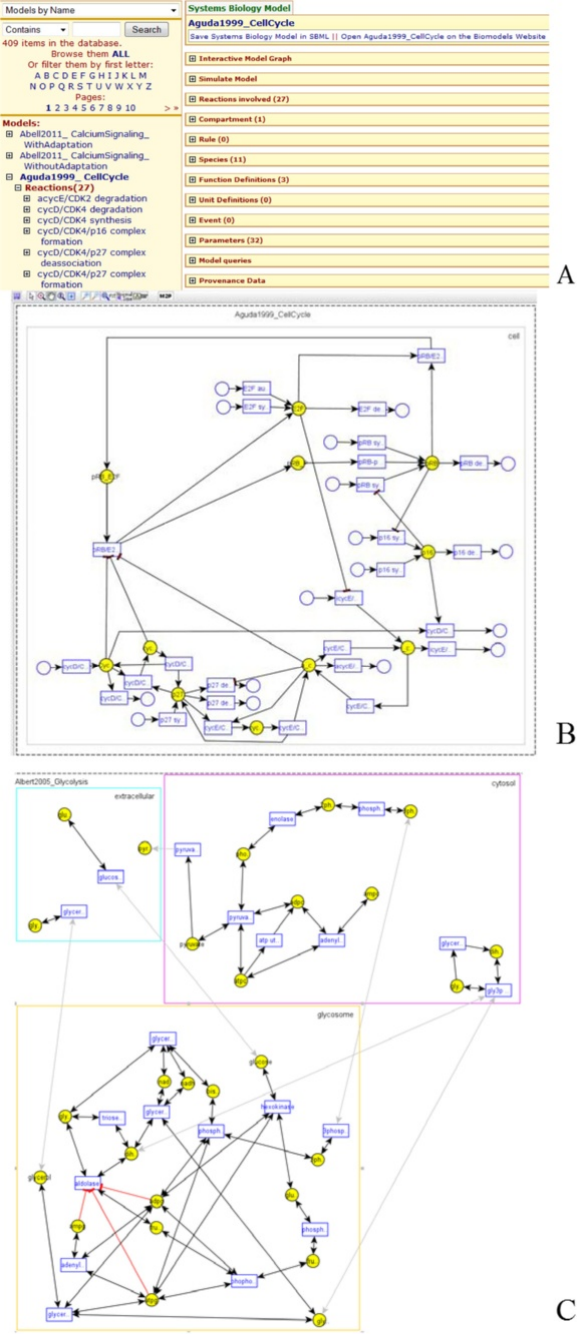
Example 2.1.

It should be noted that some of the above-listed capabilities (more specifically, browsing and visualization) are provided in some manner by original data sources, e.g., BioModels Database and KEGG. However, because of integration, PathCase-SB has the opportunity to add new features to these capabilities; e.g., see Figure 1.D that illustrates the visualization of a mapping between the modeled network of a systems biology model and KEGG pathways. PathCase-SB is solely focused on integrating systems biology data sources and providing more powerful and more comprehensive versions of these (and, into the future, possibly other new) capabilities; e.g., see visualizations in Figures 2.B and C.

Next we illustrate one possible use of PathCase-SB with an example.

**Example 1.1.** PathCase-SB user Audrey Elif has the KEGG pathway *TCA Cycle* in her mind, and wants to search PathCase-SB for BioModels Database models which contain this pathway.

**Step 1.** Audrey Elif locates two options: (a) Access PathCase-SB Browser interface and obtain a list of models using the functionality of “browsing models by KEGG pathways”, as shown in Figure 1.A, and (b) Access PathCase-SB Built-in query interface and obtain a list of models using any of the following two queries: “Find models that contain reactions of a given pathway”, as shown in Figure 1.B; or “Find models that contain metabolites of a given pathway”.

**Step 2.** Audrey Elif chooses one model from the results of step 1, and checks the visualization of the chosen model, as shown in Figure 1.C. Then, she uses ‘M2P’ tool to see the mapping between the TCA Cycle and the chosen model, as shown in Figure 1.D.

**Step 3.** Using the visualization of the mapping, Audrey Elif chooses two (or, perhaps up to four) models which have similar mappings. Then, she uses the PathCase-SB SimCom tool to inspect simulation results for the chosen models, as shown in Figure 1.E.

In summary, the premise of PathCase-SB is that performing systems biology research can be made more effective and easier by the use of an integrated environment for regulatory metabolic network models and metabolic pathways resources, and by new computational tools.

## Implementation

Next, we summarize the implemented capabilities of PathCase-SB in more detail.

### Model+metabolic network visualization capabilities

PathCase-SB *Visualization Interface* is powered by PathCase-SB *Graph Viewer* (a client-side JAVA applet) that produces interactive pathway graphs, biochemical network graphs modeled by systems biology models, or both, with various mappings between them. The visualized model network and/or pathway can be manually or automatically rearranged, zoomed in/out, panned, expanded/collapsed, queried from, saved locally as JPEG file, and studied in detail. The Graph Viewer, when accessed from different places within PathCase-SB, has many different legends, basic controls, and toolbar capabilities. Visualizations include (i) full PathCase-SB metabolic network (in multiple condensed/expanded forms), individual pathways, metabolic sub-networks, and networks of systems biology models, (ii) results of queries that return metabolic (sub)networks, or (iii) metabolic networks of user-uploaded models.

### Model + metabolic network browsing capabilities

PathCase-SB *Browser Interface* provides a variety of browsing-based mechanisms for users to access PathCase-SB database, starting from a basic overview that lists the entities in the database to hierarchically drilled-down levels that include, among others, reactions, species, and compartments. A multi-faceted view of the database is provided, which allows users to access the biochemical information with distinct focus points. As an example, researchers can browse models by their corresponding pathways, studied organisms, or relevant Gene Ontology GO [20] terms (e.g., for an enrichment pre-study). Each browser item is linked to an information-rich “details page” that organizes (i) lists of participants and their roles in each model entry, and the kinetic models of the corresponding biochemical reactions and their parameters, (ii) gateways to interactive graphical tools and interfaces (e.g., simulation and visualization engines), (iii) data provenance information for source tracking, and (iv) entry points to related parameterized queries for a customized and focused study of the underlying data. In addition, the PathCase-SB Browser Interface provides to users

· An embedded in-place keyword search facility with paged result listings,

· Relationships between BioModels Database models and ontologies (e.g., the Gene Ontology and the EC (Enzyme Commission) number ontology [21]), and

· Biological compartment-based relationships between different models. The idea is to allow modelers to see the listings of models that capture biological networks.

### Model + metabolic network querying capabilities

PathCase-SB currently allows *built-in* (i.e., predefined) queries involving models *and* other database objects. For the time being, we have chosen not to implement ad hoc queries, i.e., queries constructed by the user during a query construction session (such as the *Advanced User interface*[22] of PathCase [14]). Built-in queries can be characterized as a small set of popular queries provided through very simple user interfaces. PathCase-SB queries are grouped into

· Model queries (e.g., such as “*Find models and their compartments containing a given species in a given organism”*),

· Pathway queries (e.g., “*Visualize a set of pathways”*),

· Reaction queries (e.g., “*Find models containing different expressions of metabolic flux associated to the same reaction”*), and

· Species/molecule queries (e.g., “*Find species that are n-step downstream/upstream from the species of a given model”*).

All built-in queries are made available through web services so that third-party applications can directly send their query execution requests to the web services, execute the requested PathCase-SB built-in query, and receive the execution results as an SBML (for a model-based output) or as a BioPAX [23] (for a metabolic network graph output) document. Such an approach promotes open data exchange, and is beneficial to other tool builders.

### SBML-based and comparative model simulation capabilities

PathCase-SB *Simulation Interface* allows users to either directly use SBML files of models in PathCase-SB database (i.e., the *SimCom tool*) or to upload their own SBML files of models (i.e., the *iModel Tool*), and simulate them in a comparative manner. For the simulation, RoadRunner [24,25], a high performance cellular network simulation service, is used through the Systems Biology Workbench [26] application programming interface (API). A third party library, called ZedGraph [27], is employed to render the simulation results as a graph.

In general, multiple models exist for the same pathway. Therefore, side by side comparisons of model simulations for the same pathway can lead researchers to observe similarities and differences between models. The *SimCom tool* provides the functionality to simulate up to four models in the same pathway side by side (in new pop-up windows) from PathCase-SB web site. Users can simulate a given model via (1) changing values for parameters, (2) selecting and changing initial concentrations or amounts of species and boundary species, (3) selecting metabolic fluxes to plot, (4) changing start and end values of time period, (5) changing tolerance values for both absolute tolerance and relative tolerance, (6) changing the number of data points to plot on to the graph, and (7) adding experimental values. Input for experimental results are manually editable on the field specified for users. Users can also find the model details (such as the version of the model, notes by the author and units) at the top of the simulation page. Finally, simulation interface is also fully-integrated with model detail pages to simulate a particular model right in-place, while concurrently browsing the details of the model.

### Capabilities to upload user models specified in SBML and to simulate them

The *iModel tool* allows users to upload their own SBML models onto the PathCase-SB web site to simulate. Uploaded models are parsed by PathCase-SB *SBML Parser*[28] which uses libSBML [29,30] library in the backend. After being parsed, the model is stored in a separate temporary database (which is emptied later for privacy and copyright protection purposes; therefore the uploaded models are not kept in the database), and input to the iModel tool. Currently, the iModel tool accepts only XML file types of up to 500KB in size to upload. The iModel tool informs the user if the uploaded model has errors, and whether or not it can be parsed correctly.

### Capabilities to compose new models from existing models

PathCase-SB Model Composition Interface allows semi-automatic merging of two existing systems biology models specified in SBML format, allowing users to (i) select and upload two models (either from PathCse-SB or from the user’s own computer), and view them via two friendly interfaces, namely, Tree View and text View interfaces, (ii) iteratively modify and view the uploaded SBML models to be merged, (iii) execute an *AutoMerge* of the models, and, if there are issues or problems during the auto-merge, (iv) manually intervene and revise the SBML specifications of either the merged model or the models being merged, and then, if needed, repeat steps (i)-(iii).

### Capturing and maintaining provenance data

Provenance, also called lineage or pedigree, is defined as metadata that tracks the steps of data derivation, which adds value to the data itself [31]. With our use of other web-based data sources, providing provenance of data in PathCase-SB is a necessity. PathCase-SB provenance is a subset of provenance information provided via BioModels Database, provided only as a one-stop access convenience to PathCase-SB users: (i) *Model related information*: Creation date, Modification date, Notes of the authors and publication id are stored in PathCase-SB database; (ii) *Author and publication related information*: Author-related information (e.g. name, affiliation and contact) is stored in the PathCase-SB database. However publication-related information (e.g. title, keywords, references etc.) is not stored in the PathCase-SB database. Instead users are referred to European Bioinformatics Institute’s CiteXplore service [32]. CiteXplore provides searching facilities on a comprehensive biological literature database that is curated using multiple sources. We link to CiteXplore using the PubMed ID (PMID) of the paper. PMID is provided as a MIRIAM URN in the SBML file.

## Results and discussion

In the next subsections of this paper, we present (a) the user interfaces of PathCase-SB, namely, the Visualization Interface, the Browser Interface, the Querying Interface, the Simulation Interface, and the Provenance Tool, (b) the issues and problems we have encountered in building the interfaces, and how we have solved them, and (c) evaluation studies of the interfaces, whenever applicable. Finally, we briefly compare the user interfaces of existing systems biology and metabolic pathway data sources with PathCase-SB user interfaces.

### Visualization interface

All visualization functionalities are provided on client machines, and with no server-side intervention or communication, allowing for a highly scalable PathCase-SB. The Visualization Interface (i) comes embedded into the corresponding model/pathway pages, and does not require any separate installation effort (a manageability convenience for users), and (ii) provides a platform-independent access regardless of the underlying operating system or browser differences on user machines (presently optimized for Internet Explorer, Firefox, Safari, and Chrome browsers).

### Graph viewer

The Visualization Interface employs a Graph Viewer that runs on the client side. The Graph Viewer, accessed from different places within PathCase-SB, has many different legends, basic controls, and toolbar capabilities. The Graph Viewer is employed by (a) the *Browser Interface* (appears as a menu item at many places with the name "Interactive Model/Pathway Graph"), (b) *Built-In Queries* (by each query that produces a metabolic sub-network), and (c) the *iModel Tool* (biochemical networks of uploaded user models). The Graph Viewer controls and capabilities are explained in depth in PathCase-SB help pages (as video documents), and will not be repeated here.

The interactive model graph shows species, reactions, compartments and the relations between them in the model. In a model with multiple hierarchically-arranged compartments, the hierarchy is explicitly visualized. Species and reactions are shown in compartments that they belong to; and roles of species in reactions are visualized with directed/undirected edges between nodes representing the species and the reactions they are involved in.

**Example 2.1.** Figure 2.A shows the details of the systems biology model Aguda1999_CellCycle (BIOMD0000000169) from BioModels Database; Figure 2.B shows the visualization of the same model. Finally, the model Albert2005_Glycolysis (BIOMD0000000211), shown in Figure 2.C, has three compartments, namely, cytosol, glycosome, and extracellular space. Next we list some of the salient features of the Graph Viewer.

#### Visualization simplifications

Some models include a large number of species and reactions, and the names of species and reactions are extremely long. To keep visualizations uncluttered, the Graph Viewer (i) uses truncation of those names (Full names and related information are also available: to see detailed information in a node, i.e., kinetic law of a reaction, the user simply moves the cursor over the node’s visualization), and (ii) species that participate in many reactions (e.g., H_2_O, ATP, ADP, etc.) and their connectivity information within the network can be elected not to be visualized. We name such species as *common species*.

#### Mapping models to pathways

For those models that are related to a pathway, the Graph Viewer provides the mapping between the model network and the pathway by displaying both side by side, and highlighting species in the model and the molecular entity in the pathway together with corresponding qualifiers (such as *is-part-of*), provided via the Graph Viewer tool named *M2P* (Model-to-Pathway). As examples, see (a) Figure 1.D and (b) the model network of the BioModels Database model *Singh2006_TCA_Ecoli_glucose* (BIOMD0000000222), which is a version of the *TCA Cycle* pathway: one can visualize (i) the relevant reaction-only mapping information, (ii) the mapping between species of the model network and metabolites of the pathway.

### Layout manipulations

Model network or pathway layout is important for helping users locate species and reactions. The Graph Viewer allows the user to manually”touch” and adjust the layout, and then to save the revised layout into the PathCase-SB database. When a user reopens the layout later, the saved layout is displayed by default. To keep the layout consistent and meaningful, currently, this functionality is only open to those who request it, and not available to public.

### Browser interface

PathCase-SB Browser Interface works with popular internet browsers, and is specifically tested for Internet Explorer, Firefox, and Chrome. The browser interface is built primarily for viewing the information related to

· Systems biology models from multiple sources (currently, only BioModels Database, and, in the future, CellML model repository), in conjunction with metabolic pathway fragments from KEGG that these models employ, and

· KEGG Metabolic pathways, in conjunction with models of BioModels Database, with a focus on presenting the relationships that exist between the various entities of the two types, i.e., models and pathways. A more detailed description of how these relationships are imported and captured in PathCase-SB database is presented elsewhere [2].

The browser Interface also presents to users

· Relationships between models of BioModels Database and ontologies such as the Gene Ontology, and the EC (Enzyme Commission) number ontology, and

· Biological compartment-based relationship between different models. The idea is to allow modelers see the listings of models that capture biological networks within the same compartment (e.g., say, liver cytosol) to help them with model composition operations, as summarized in the Model Composition Interface section.

Currently, PathCase-SB only allows browsing systems biology models from BioModels Database. In the near future, we will add browsing features for other systems biology sites, such as CellML model repository. The browser interface allows users to browse the database based on different “views” of data. Each view is a tree starting with the root as the chosen node, and t can be expanded to arbitrarily many levels. Each node represents an entity that can be expanded, which brings up other nodes under it, if any exist. Clicking to the text on the entity brings up a display on the right hand side that gives more detailed information about that entity. The left hand nodes are called Browser items, and the right hand page, called the Details page, presents the details of the browser item.

The browser interface currently provides the following browsing capabilities:

· BioModels Database data browsing starting with Models (covered as a separate section below), Reactions (covered as a separate section below), Compartments (covered as a separate section below), or Species (covered as a separate section below), and

· KEGG data browsing, namely, Pathways, processes, and molecular entities. We do not cover this part since it is borrowed directly from original PathCase [14].

· Gene Ontology (GO) terms browsing, and locating models that model enzymes/genes with the listed GO term (covered as a separate section below),

· EC (Enzyme Commission) number taxonomy browsing (covered as a separate section below), and locating models that model enzymes/genes with the listed EC number,

· Browsing the organism hierarchy provided by BioModels Database models (covered as a separate section below), and locating models of the selected organism.

PathCase-SB database can also be searched without browsing through the database. Different types of entities in the PathCase-SB database can be searched by name. There are four options that allow searching for a specific entity given its whole name or parts of the name.

### Querying interface: built-in queries

PathCase-SB is designed for users to pose built-in (i.e., predefined) queries involving models and other database objects. We define built-in queries as a small set of queries that are the most popular ones. Clearly, this is a subjective definition. So, as we have done with PathCase [14], we have selected built-in queries of PathCase-SB from our experience, which evolve as needed. The idea is that built-in queries are so useful and so common that, for ease of use, they are provided through very simple user interfaces. In addition, for all fully implemented queries, the queries have visualization functionalities for the returned models/pathways. Note that some of the built-in queries are pathways-only queries from PathCase, and, provided only as a convenience. Next we discuss the implemented built-in queries

### Queries

The fully implemented PathCase-SB queries are grouped into (1) Model queries (six queries); (2) Pathway queries (two queries); (3) Reaction queries (eight queries); (4) Species/molecule queries (four queries).

### Model queries

**Query 1.** Find models corresponding to a given reaction in a given pathway. This query involves information about both models and the pathways, referring to reactions modeled by a model and the corresponding KEGG reaction in specific pathways. Two alternative mappings can be used during the query processing: (i) model-to-pathway, and (ii) reaction(BioModels Database)-to-reaction(KEGG). For now, we assume reaction-to-reaction mapping is used, and the user specifies a process of a particular pathway as query input.

**Figure 3:**
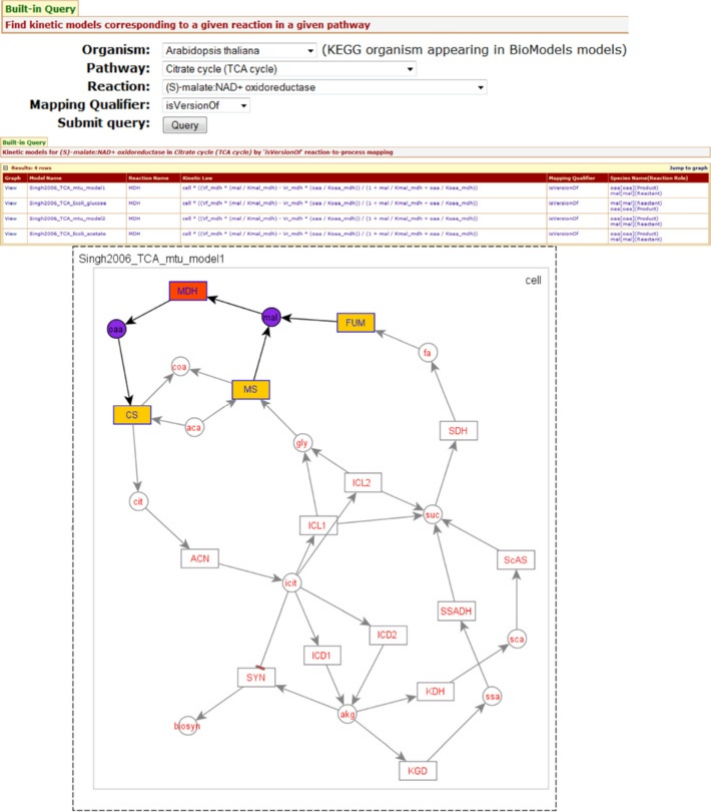
**An example input and output of built-in query*“Find models corresponding to (S)-malate:NAD+oxidoreductase in Citrate cycle (TCA cycle) by 'isVersionOf' reaction-to-process mapping”***.

**Example 4.1.** Figure 3 illustrates a sample query type 1 for model queries: “*Find models corresponding to (S)-malate:NAD + oxidoreductase in Citrate cycle (TCA cycle) by 'isVersionOf' reaction-to-process mapping*”.

**Query 2.** Find models having molecular species in a given domain (compartment, organism). Assuming the user chooses to specify compartment C, this query returns both textual and graphical information: (i) a four column table < graph-view, model-name, {set of species in compartment C}, compartment C>, and (b) the graphical view of the first model returned. The species of the model are red-colored in the graphical view.

**Query 3.** Find models and their compartments containing a given species in a given organism.

**Query 4.** Find models that contain reactions of a given pathway.

**Query 5.** Find models that contain metabolites of a given pathway.

**Query 6.** Find models containing different expressions of metabolic flux associated to the same reaction.

### Pathway queries

**Query 1.** Find pathways within a given number of steps from a pathway.

**Query 2.** Visualize a set of pathways.

### Reaction queries

**Query 1.** Find reactions (and their kinetic equations if exist) that are n-step downstream or upstream from the reaction of a given model.

This query requires mappings between species of different models. This query is evaluated by assuming that species that are mapped to the same KEGG molecular entity are the same entities. Then, shared species between models can be used to construct a model network, on which the query is executed.

**Figure 4:**
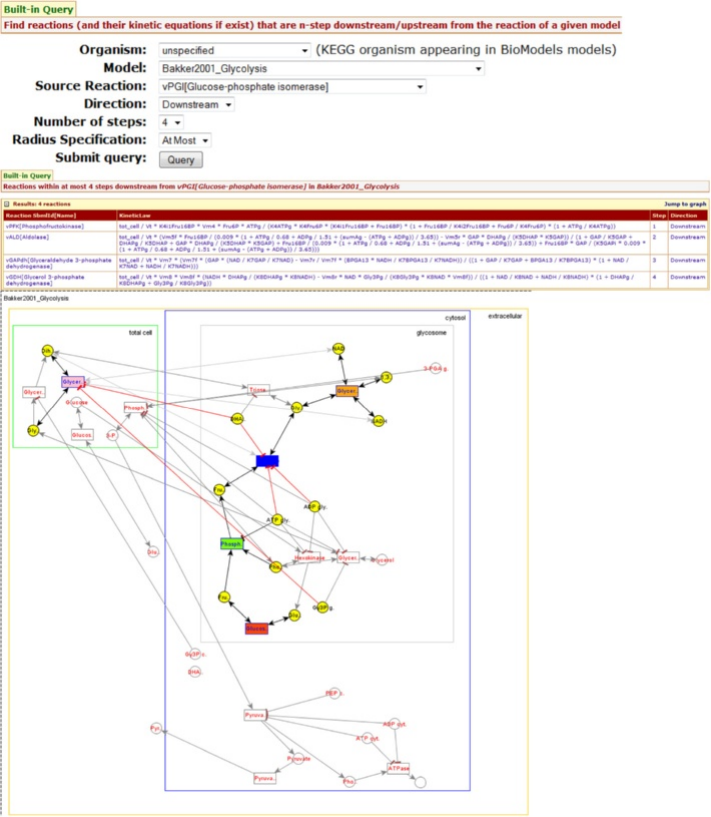
**An example of built-in query*“Find reactions within at most 4 steps downstream from vPGI[Glucose-phosphate isomerase] in Bakker2001_Glycolysis”***.

**Example 4.2.** Figure 4 illustrates a sample query type 1 for reaction queries: “*Find reactions within at most 4 steps downstream from vPGI[Glucose-phosphate isomerase] in Bakker2001_Glycolysis*”.

In addition, the other seven reaction queries of PathCase-SB are: (1) Find reactions within a given number of steps from a reaction in a pathway; (2) Find reactions within a given number of steps from a molecule in a pathway; (3) Find reactions within a given number of steps from a reaction in the metabolic network; (4) Find reactions sharing activators and inhibitors with a reaction in a pathway; (5) Find reactions with the given number of molecules in a specific use; (6) Find reactions involving exactly one substrate and one product; (7) Find reactions involving a molecular entity in a pathway.

### Species/molecule queries

**Query 1.** Find molecular species that are n-step downstream/upstream from the molecular species of a given model.

Note that this query can only be answered within a specific systems biology model. Performing the same query on a network of models requires mappings between species of different models. This query can be accomplished, if species that are mapped to the same KEGG molecular entity are considered as the same “entities”. Then, shared species between models can be used to construct a model network, on which this query can be executed with possible results from multiple models.

In addition, the other three PathCase-SB species/molecule queries are listed as: (1) Find pathways/reactions involving a molecular entity with a specific use; (2) Find molecules within a given number of steps from another molecule in the metabolic network; (3) Find paths between two molecules in a pathway.

### Simulation interface

Our simulation interface efforts have focused around

· Directly using SBML files as input to the simulation process, as the models are exchanged and made available in SBML and

· Integrating existing stand-alone simulation software, namely, RoadRunner [24,25], into the PathCase-SB system, as there is already a number of sophisticated and highly-capable

· Simulation software available to the research community.

In our initial simulation efforts, we employed systems biology models with different level of complexity to describe metabolic and physiological processes. All models were represented in different format using Matlab and SBML implementations. One of these models was previously developed by PathCase-SB team researchers as part of their research on bioenergetic function in skeletal muscle [33] and it is available on BioModels Database website [3]. Next, we briefly describe the simulation tools and models we have studied, followed by (a) a discussion of the problems we have encountered, (b) a description of the capabilities of simulation interface that is integrated into our system, and (c) a description of two new simulation tools which use the simulation interface.

### Existing simulators and simulator used by PathCase-SB

There exist a large number of available simulation tools that can potentially be utilized by PathCase-SB Simulation Interface. For the selection of tools to work with many alternatives, we specify two requirements that are critical to accomplish our goals within this project:

· A simulation tool should be easily portable to our PathCase environment, which is coded mainly in C# and ASP.NET, and

· The simulation tool should support events in SBML, as our in-house models frequently make use of events as part of their specification.

**Figure 5:**
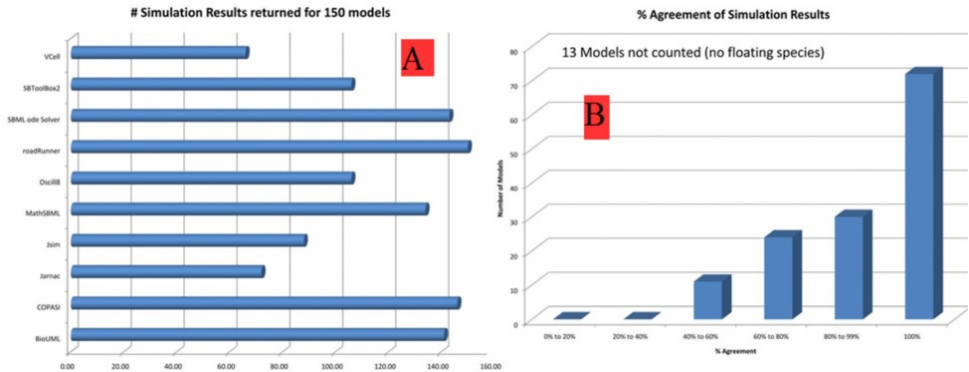
Simulation tools.

After working with RoadRunner, CellDesigner [34], and MathSBML [35], we decided on RoadRunner which provides up to date .NET compatible wrapper classes in its API for remote procedure calls. From the bar chart in Figure 5.A, it is seen that RoadRunner returns the largest number of simulation results for 150 models which are chosen from BioModels Database. Figure 5.B displays the reliability of the simulation results of the BioModels Database models excluding 13 models and floating species (i.e., an SBML species element without a boundary condition). Since RoadRunner is capable of simulating the widest range in terms of different type of models among the compared simulators, it fits our needs.

#### Simulation interface test results

The simulation interface was tested with four models with different levels of complexity in describing metabolic reaction and transport processes in physiological systems. Each system is described by a set of ODEs that represents the mathematical model. To simulate the kinetic processes of the system, the mathematical model is solved using RoadRunner. In general, the accuracy of the model simulations obtained with the simulation interface is the same of that obtained with other SBML simulators.

### Simulation interfaces for biological models

#### Overview

There are about tens of different simulation software for SBML documents [36]. From this list, some of the software is not free; some of them are working only on Windows operating system; and some of them do not provide all the capabilities needed by users. In addition, installing the correct updates of the installed software to the client is a difficult task. On the other hand, a web interface is updated on the server side, and, hopefully, always up-to–date; and, users can access the tools from everywhere with no machine compatibility issues.

**Figure 6:**
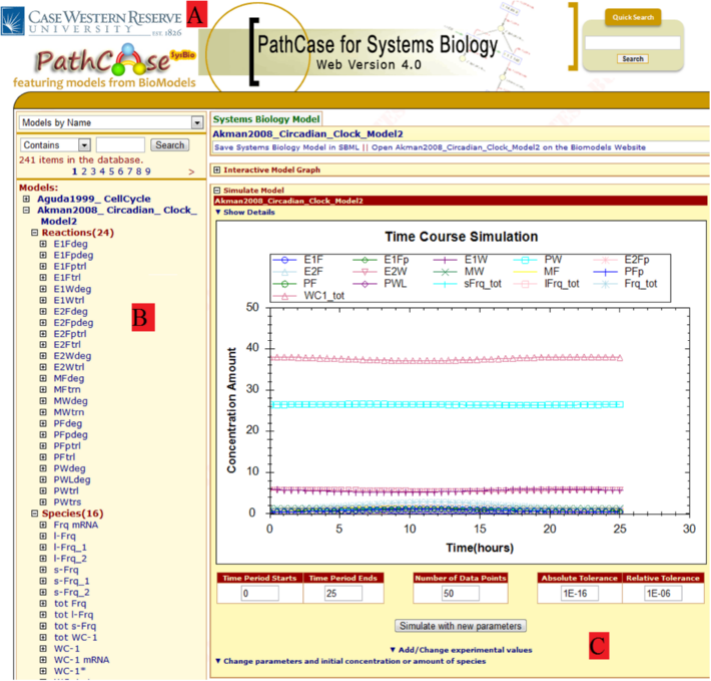
**Integrated biological model simulation interface in PathCase-SB.A**) PathCase Systems Biology 4.0 Header for PathCase-SB. **B**) Model list by name. **C**) Simulation results for the model.

For PathCase-SB, the web based simulation interface is built and integrated to the PathCase Systems Biology web site. Model simulation is integrated into many places on the PathCase Systems Biology web site, and Figure 6 shows its integration to "Models by Name" search as an example. From the left hand side tree list (i.e., the Browser Interface), the user can click on any model which is already parsed into the database to simulate the chosen model. Once the model is chosen, on the right hand side, the user can see the simulation results within "Simulate Model" collapsible panel.

In PathCase-SB simulation interface, users can re-simulate the model via:

· changing values for parameters,

· selecting and changing initial concentrations or amounts of species and boundary species,

· selecting metabolic fluxes to plot,

· changing start and end values of time period,

· changing tolerance values for both absolute tolerance and relative tolerance,

· changing the number of data points to plot on to the graph,

· adding experimental values, and

· observing the results of the new simulation.

Input for experimental results are also manually editable on the field specified for users. Users can find model details (such as the version of the model, notes by the author, and units) at the top of the simulation.

Next, we explain each of the above-listed features in detail.

### Details of SBML model used

Each and every SBML model has an SBML level and version number which specifies the structure and capabilities of the SBML file. Some parsers cannot parse, or parse specific levels and versions incorrectly; therefore, this information is crucial for the researcher to make sure that a compatible (or newest) SBML version and level are used. At the time of this writing, the simulation engine used within the simulation interface is compatible with versions 1, 2, 3 and 4 of Level 2 SBML models. Since units for time scale are usually defined in unit definitions in SBML files, we automatically parse the time information and write to the plot. Nonetheless, substance scale can be more complex to parse automatically; therefore we give all other units in the units section of details. In addition, SBML models contain a “notes” section for modelers to add detailed notes which generally contain personal explanations for the model, details of their model, and the citation information. If the modeler specifies the information via hyperlinks to other pages, these URLs will be active for redirection in the notes section. All of this information is available to the user in the simulation interface once s/he clicks the "Show Details" link at the top of the simulation graph.

### Basic settings for simulation

From the simulation interface, the user can define custom values for basic simulation settings which reside underneath the plotted graph.

### Parameters and initial concentration or amount of species

Once the user expands "Change parameters and initial concentration or amount of species" link, a list of options for available species, boundary species, parameters, and metabolic fluxes appears. The user can modify the parameter values from the left column parameters list, and also the units for these values (shown next to each field in parenthesis).

### Metabolic fluxes

In addition to species, boundary species and parameters, users can see the metabolic fluxes on the simulation graph. Users can select and deselect to show or hide the metabolic fluxes on the simulation graph. In the beginning, by default, none of metabolic fluxes are checked, and this means none of them are plotted in the simulation graph.

### Experimental values

Researchers may want to compare their data (possibly prepared after conducting many experiments) with a currently curated and accepted model. This way, the overlaps between experiment data and the original model simulation data can be compared easily. In the PathCase-SB simulation interface, the user can enter the experimental values into a large text field by clicking to the "Add/Change Experimental Values" link. Simulation can be rerun via clicking the "Simulate with new parameters" button. To differentiate easily, only experimental data is plotted in red in the simulation graph by default.

### Technical details

Implementation of the simulation interface is done with ASP.NET Framework using the C# language. User interface itself uses the asynchronous JavaScript and XML (AJAX) technology in order to achieve better performance and more friendly user experience. For the simulation in the backend, the RoadRunner application programming interface (API) is used. A third party library called ZedGraph is used to plot the results onto a graph. The modular implementation (built as a user control) of the simulation interface provides easy integration to other parts of the PathCase Systems Biology web site such as SimCom and iModel tools which are discussed next.

### PathCase-SB simulation tools

#### Simulate and compare biological models side by side (SimCom)

In general, for the same pathway, there are multiple models. Therefore, side-by-side comparison of these biological models for the same pathway can allow researchers to identify the main similarities/differences between similar models. The SimCom tool provides the functionality to simulate up to four models in the same pathway side-by-side (in new pop-up windows) from PathCase-SB web site.

From the main web page of PathCase Systems Biology web site, users can click on the "SimCom" tool link from the left bottom corner to start using the SimCom tool. Once the user selects a pathway from the dropdown list, a model list for the selected pathway is loaded under it. In square brackets, organism information for each model (whenever available) is displayed next to each model. After selecting models to compare, the user can simulate all selected models side by side; see Figure 1. as an example. For each selected model, an “independent” fully functional simulation interface (as described in the previous section) is loaded in new pop-up pages side by side. Since the simulations are independent of each other, the user can close one of them without changing the state of the other simulations, and continue modifications on the open ones.

### Simulate user uploaded biological models (iModel)

SBML format is used to share and examine a biological model. In general, even for a quick look at simulation results of an SBML file, users need to install software to their client machines. Only after installing and setting up the compatible software, users can upload their model and see the simulation results. There may be few problems here: finding the correct/compatible client software is not always an easy task; and, keeping the client software up-to-date is another task.

By using the iModel tool, users can upload their own SBML models into the PathCase-SB site to simulate. Uploaded models are parsed with the PathCase SBML Parser which uses libSBMLlibrary in backend. After being parsed, the uploaded model is stored in a separate temporary database which is emptied after use. Therefore, the uploaded models are not maintained or kept in our original PathCase-SB database for privacy issues. Currently, the iModel tool accepts only XML file types of up to 500KB in size to upload. If the uploaded model has errors and cannot be parsed correctly, iModel will indicate to the user that the model is incorrect.

From the main web page of PathCase-SB web site, users can click on the "iModel" tool link to start using iModel tool. By using the "Choose File" button, users browse their machine, choose the SBML model file to be uploaded, and then click on the “Upload My Model” button. If the model is uploaded and parsed successfully, the “See Uploaded Model” button appears, and the upload panel is hidden. Once the user clicks on the "See Uploaded Model" button, an independent pop-up window opens up with the fully functional model simulation interface that is described in previous section. In order to upload another model without closing the currently uploaded model, users can use the “Upload New Model” button to be able to bring the upload file panel back.

In summary, currently, the PathCase-SB simulation interface uses RoadRunner as its simulation engine. RoadRunner simulator is in active development (with issues and bugs being solved) and one of the reliable simulators, as of January 2010. Nonetheless, since PathCase-SB simulation interface is like an overlay on top of RoadRunner, we do have the ability to move to another simulator (e.g. Jarnac, Oscill8, etc.) in the future, if it provides better simulation results as compared to RoadRunner. The PathCase-SB simulation interface is tested with the recent versions of Microsoft Internet Explorer, Mozilla Firefox, Google Chrome and Apple Safari web browsers.

### Model composition interface

There are already few thousands of verified and curated small sized systems biology models, available mostly at BioModels Database, and CellML model repository, and there is an emerging need to combine existing models, and to capture larger biological networks. In addition, researchers would like to test their own private computational models by composing them with verified computational models in model repositories. There are three major bottlenecks for creating a large systems biology models.

· As of now, there is no standardized approach accepted by the community to merge multiple SBML models into a larger one, even though there is an effort to standardize model composition [37].

· Designing larger computational models is usually more difficult than building models for sub-networks of specific biological networks. And

· Developing large and highly complex biological models is not well-aided by integrated free biological modeling software.

The PathCase-SB Model Composition Interface [38] provides a three-step model composition process for computational models defined in SBML format.

**Step 1:** The user selects the models to be combined. PathCase-SB Composition Interface provides an upload functionality through which the user can upload his/her own SBML model from their computers. In addition, users can choose models to merge from the already parsed SBML models on PathCase-SB. To help researchers with model selection, PathCase-SB model composition interface provides “similarity indices” between two pre-parsed models [38] in order to aid the researchers in picking the most appropriate model for their needs.

**Step 2:** In a simple and user-friendly manner, the interface allows users to manually match model element names before the semi-automated merge takes place. After completing the manual matching step, the user clicks on the button “Done with Manual Matching. Start Composition Interface”, and the *AutoMerge* algorithm is executed. The *AutoMerge* algorithm automatically matches compartments, reactions, and species of the two models being composed. Matching basically works on the “id” attribute of SBML element. While comparing two SBML elements in the XML file, strictly following name matching conventions increases the chances of matching SBML elements in the composed model. For instance, in *Akman2008 Circadian Clock Model2* SBML model, there is a species element which has id *MF* and name *Frq mRNA.* When composing this model with another model, if the same species are named the same, automated algorithms can easily match these two species. In addition to name matching, *AutoMerge* also checks for matches in (i) compartment attributes and (ii) *sboTerm* attributes, if available, for species. For reaction elements, attribute information about *name*, *reactants* and *products* for each reaction of the first SBML model are compared and matched to those of reactions in the second SBML model in order to identify whether the two SBML models share common reactions. Compartment matching is based on the name attribute.

**Step 3:** Users employ the tools of the interface, and edit the merged model via the “tree list view” or “textbox view” interfaces, and update the resulting model. *AutoMerge* applies a number of merge integrity rules [38], and, as a result, some parts of SBML documents may not merge correctly. In that case, the model composition interface provides a list of *Warning Messages*, to be manually resolved by the user. Some XML attributes such as *unit*s, *initialAmount*s, and *stoichiometry* may not match even when the *id*s and *name*s of XML elements do match. In addition to attribute value conflicts, *id* conflicts are also possible, and manual intervention there is also needed. For more details, see Coskun et al., 2011 [38].

### Provenance tool

In this section, we summarize the concept of provenance, and then describe the current state of the PathCase-SB Provenance Tool. In the first section below, we provide the classification of PathCase-SB provenance tool in the provenance taxonomy. Then, we present details about the model and implementation of the tool.

Provenance is defined as metadata that tracks the steps of data derivation, which can add value to the data itself [39,40]. Also called lineage or pedigree, provenance is an important research area. Scientists, who base their research on the results of the previous works, seek a confidence on the reliability of what has been proposed [40].

In the context of PathCase-SB, it is very important for a user to assess the trustworthiness of a provided systems biology model. The creators of a given model, the publications that have led to the model and the references of the model are crucial metadata about the model. Hence, providing provenance data about systems biology models is an important task.

### PathCase-SB provenance classification

**Figure 7:**
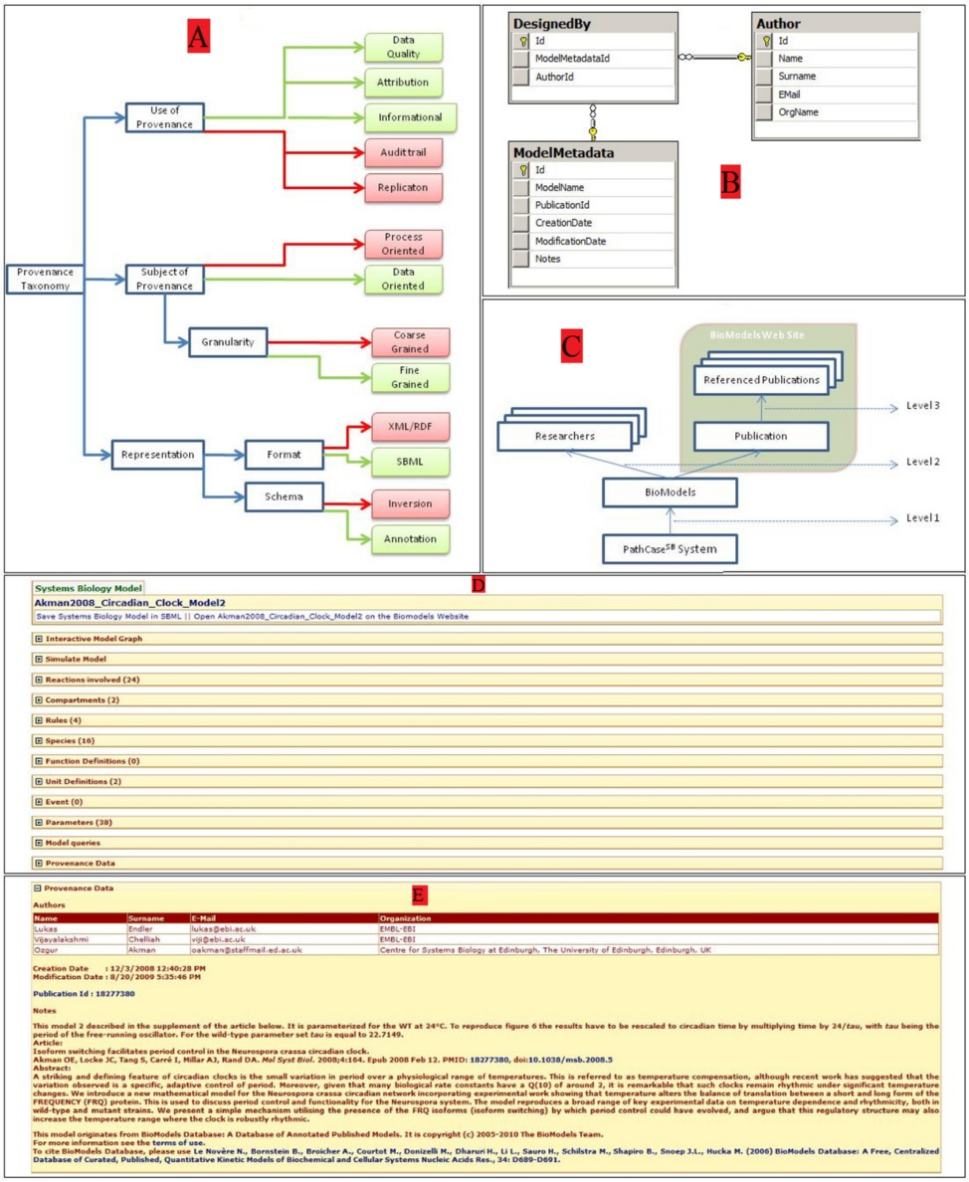
**Provenance System Design.A**) The place of PathCase-SB Provenance Tool in Provenance Taxonomy. **B**) Database tables for provenance data. **C**) Provenance data model. **D**) Collapsed Provenance Panel in the model page. **E**) Provenance Data for the model *Akman2008*_*Circadian*_*Clock*_*Model2*.

We make use of the provenance taxonomy given by [39] to characterize the PathCase-SB provenance tool. We briefly explain terminology used in [39]; the reader is referred to the original work for further information. Figure 7.A shows where PathCase-SB fits in this taxonomy. There are five types of usages for provenance: (i) Data Quality: The reliability or the quality of the data is defined. (ii) Audit Trail: Errors in the generation process is detected and the audit data is tracked. (iii) Replication: Provenance is used as a log to replicate the data in the future. (iv) Attribution: Provenance is used for copyright and citation of the original data. (v) Informational: Provenance is used to provide generic information about the data. We concentrate on *data quality* provenance in PathCase-SB since the models in the database belong to other researchers, and PathCase-SB simply displays what is submitted. Therefore it is important to display where a model comes from for users who seek reliability. *Audit trail* provenance does not apply to PathCase-SB as we do not know about the data generation process. PathCase-SB does not replicate the data; so *replication* provenance is also not relevant for PathCase-SB. *Attribution* provenance does apply to PathCase-SB as a significant part of the information displayed by PathCase-SB belongs to others, and PathCase-SB cannot act as if it owns the data. *Informational* usage also applies, as models in general are not fully self-explanatory, and comments from the creators are useful sources to understand the model. Provenance can be process or data-oriented. *Process-oriented* provenance focuses on the processes that generate the data versus the *data oriented* provenance, which focuses on the final products (data) [39]. PathCase-SB is clearly interested in providing *data oriented* provenance as it collects information about the models themselves, and not the generation process. The *level of granularity* in PathCase-SB is at the model level as it collects provenance about each model. Therefore it is *fine-grained*. Representation of provenance is classified into Scheme and Format [39]. There are two types of methods to formulate the scheme: Annotation and Inversion. In the *annotation* method, the provenance data is stored as annotations about the data, in contrast to the *inversion* method, which uses techniques to invert queries that are used to obtain products in order to reach the source. PathCase-SB clearly fits into the *Annotation* method. The typical format used for annotations transfer and storage [39] is XML or RDF. The models used in PathCase-SB (from BioModels Database) are in the form of SBML, which is a derivation of XML, and the provenance data is embedded in SBML documents.

### PathCase-SB provenance: data storage, data model, and implementation

SBML documents obtained from BioModels Database [3] have two special elements (tags) that provide provenance data about the model itself. The < annotation > element gives information about the creators of the model (name, surname, e-mail address and the affiliated organization), creation and modification dates of the model and the PubMed ID (PMID) as a MIRIAM URN. The < notes > element stores the comments of model creators as plain text that describes the model generation process and any other information deemed necessary by model creators. PathCase-SB maintains three provenance-related tables, as shown in Figure 7.B. *Author* table stores the author’s name, surname, e-mail address and organization. *ModelMetadata* table stores the model’s id, creation date, modification date, notes of author and the publication id. And, *DesignedBy* table stores the model ids and the corresponding author ids, which are related. PathCase-SB provenance model includes three levels of provenance data as shown in Figure 7.C. The first level is model related information: Creation date, Modification date, Notes of author and PMID. This data is stored in PathCase-SB servers. There are two layers in level 2: The authors and the publications. Author-related information is again stored in PathCase-SB database, whereas publication-related information is not and user is linked to CiteXplore [32] as explained in Implementation Section. The final level is the third level that is connected to the referenced papers. These are the papers that are being cited by the publication of the model. Users are again referred to CiteXplore for this level. As CiteXplore provides an advanced search engine and a comprehensive biological literature database that integrates many sources, we have chosen not to replicate this functionality, but to provide links to the source. PathCase-SB provenance information is provided as a stand-alone panel within the model page of the PathCase-SB system. When a model is selected, along with other functionalities for the model (such as Interactive Model and Simulation Tools), the provenance panel is placed at the very bottom of the right-hand side frame. Figure 7.D shows the list of specifications provided for the "Akman2008_Circadian_Clock_Model2" model, and the provenance panel is listed at the bottom. Clicking on the collapsed panel shows the provenance data about the model. Figure 7.E displays the provenance tool panel for the model in Figure 7.D. First, data about the creators of the model is listed. For each author, we list the name, surname and the organization of the author. After the authors, the creation and modification dates for the model is listed. Publication Id field displays the PMID for each model. Clicking on this id takes the user to the page of this model in CiteXplore. Finally “notes about the model” are displayed. This is the place where the author provides supplemental information about the model. For instance, in the model of Figure 7.D, the author mentions the temperature that the model is parameterized for. The notes part is also shown within the simulation tool to provide users in-place information about the model and the simulation if it is supplied.

### Comparison of PathCase-SB with BioModels database

Below, we briefly compare (tested as of February 2012), the BioModels database and PathCase-SB in terms of the model repository, simulation, visualization, model search and composition, and other features.

### Model repository

BioModels Database has 409 curated and 420 non-curated models(as of April,2012). The curated models have been thoroughly curated, and model elements have been annotated with terms from controlled vocabularies as well as links to relevant data resources. For non-curated models, the syntax has been verified, but the semantics remains unchecked. Curated models can be downloaded in SBML L2 V4, or SBML L2 V1 (auto-generated), or SBML L2 V3 (auto-generated). Other formats (auto-generated) are also provided, i.e., BioPAX (Level 2), BioPAX (Level 3), Octave (m-file), PDF, SciLab, VCML(VCell), XPP.

### PathCase-SB has 409 curated models(as of April, 2012), and user can export models in SBML L2 V4

Both BioModels Database and PathCase-SB have “browse models by name” and “browse models using GO” features. BioModels Database allows user to “browse models by BioModels ID“,”by Publication ID”, “by Last Modified”. PathCase-SB allows user to “browse models by KEGG pathways”, “by Author Names” or “by Organisms”. PathCase-SB also provides an interface to browse all reactions, species and compartments of all the models.

### Model simulation

Both BioModels Database and PathCase-SB allow user to select species and fluxes to display in the simulation. Also, user can change values of parameters, fluxes and species for re-simulation. Both systems export the results of the simulation (comma separated values) and have documentation on how to use the simulation interface.

BioModels Database uses two simulation tools. JWS online simulation [41] simulates models with JWS Online, which requires JRE [42] to be installed on the user’s machine. BioModels online simulation does not need JRE on the client side, but the user has to wait, and refresh the web page to see the results.

PathCase-SB works on all major browsers without installing any additional software. PathCase-SB provides visual help on how to use the simulation interface. Some other features available in PathCase-SB include: users can (i) add experimental data to the simulation results, (ii) change number of time points and absolute/relative tolerance values, (iii) upload users’ own SBML model and use the same simulation interface on it on the fly without converting the model to any other format, (iv) compare the related models side by side within separate windows easily, and (v) display SBML notes.

### Model visualization

BioModels Database provides “View Bitmap Reaction Graph”,”View SVG Reaction Graph” and “View Dynamic Reaction Graph” functions; however, these visualizations do not include compartment hierarchy or species with multiple roles in the reactions.

PathCase-SB visualization produces interactive pathway graphs, model graphs or both with various mappings from one to the other. The visualized model/pathway can be manually or automatically rearranged, zoomed in/out, panned, expanded/collapsed, exported locally as jpeg file, and studied in detail such as displaying kinetic law of reactions. Also, PathCase-SB allows users to change and save the layout of the visualization. Users can upload his/her own SBML model and see the visualization on the fly.

### Searching

BioModels Database searches all information related with the model, and produces a large number of output results. The modifiers includes “and” “or” “match the exact phrase”. The identifiers include “BioModels”, “Person”, “SBML elements”, “Annotation (full text)”, “Annotation(identifier)”.

PathCase-SB searches name only, but allows users to choose categories like “Models”, “Reactions”, “Species”, “Compartments”, “KEGG Pathways”, “KEGG Processes”, “Molecular”, “Organism”. The modifiers include “Contains”, “Starts with”, “Ends with”, “Exact match”. PathCase-SB highlights keywords in the search results, and counts the results according to the categories. PathCase-SB also provides built-in queries.

### Provenance

PathCase-SB provenance capabilities are a strict subset of the extensive provenance capabilities of CiteXplore [3]. Nevertheless, in order to help users to provide direct data provenance information next to models they view in PathCase-SB, the system maintains and displays limited provenance information. CiteXplore has extensive model-related provenance data. They provide the name of the person who has submitted the model, the date of submission, papers that have cited this paper, the chemicals mentioned in the paper, the abstract and the link to the full text of the paper. This information does not exist in BioModels Database SBML documents, and, therefore, it is out of reach for PathCase-SB parser. CiteXplore displays a link to the original paper in a separate field, whereas PathCase-SB can display this information only if it is mentioned in the notes of the creator.

### Composing models

BioModels Database does not have a model composition tool. PathCase-SB model composition tool helps users compose standard SBML-formatted models in a semi-automatically way.

### Other features

PathCase-SB provides browsing of KEGG pathways, processes, molecular entities, gene ontology, EC numbers and organisms.

### Comparison of PathCase-SB with other systems biology sources

The systems below have been tested in December 2011, and February 2012. Metannogen [43] is a stand-alone Java application, which needs to be downloaded to function. It can be used to annotate the existing networks or reconstruct metabolic networks. The networks can be loaded from a web address or from a local file. Three networks, namely, EHMN [44], KEGG and Recon1 [45], are provided in the demo dataset on the server. Figure of a chemical formula for each molecule is provided in the program. Reactions are organized according to pathways. Users can (i) add an XML attribute to an existing SBML file, (ii) create RDF [46] style annotation for each reaction which is then added into the existing SBML file. The network reconstruction can use the reactions in already published networks. The equation and the metabolites of the reaction can be revised. Metannogen also allows compartment information for the reactions. Finally, the result can be exported to an SBML file. There’s no visualization for the reconstructed network. Metannogen downloads and allows users to view KEGG pathways as static graph.

Ondex [47] a client application that integrates and visualizes biological data set. Ondex does not have model simulation capabilities. There’s no web-based version, and SBML version is not supported right now (in development). User can load existing networks from supported data file, or import the data, which have parsers available within the Ondex system (there are seven generic parsers including SBML and 25 database/tool-specific parsers including KEGG parser). The imported data may be visualized using Ondex (in .oxl format), which displays data as a set of linked graphs where nodes represent data objects (i.e., pathway, reaction, gene, enzyme, drug, KEGG ortholog group, etc.) and edges represent relationships (e.g., “reaction is member of pathway”, “drug is produced by reaction”, “reaction is catalyzed by enzyme”, etc.) between nodes.

WebCell [48] is a web-based application, which has network visualization, and model validation and analysis components. The visualization is also applet-based, and much simpler than PathCase. There is no kinetic law information or compartments, and nodes cannot be moved. There’s no comparison with Kegg pathways. At the time of testing, WebCell simulation tool did not work with uploaded models, but it worked well with pre-parsed models on their web site.

WebCell simulation tool allows user to (i) modify time period start value, (ii) modify absolute and relative tolerance values, (iii) modify parameter values, (iv) modify species initial concentrations, (v) add experimental values, and (vi) download simulation results.

CopasiWeb [49] provides stochastic and deterministic time course simulation (steady state, time course, metabolic control analysis, parameter scan, sensitivity analysis, parameter estimation). CopasiWeb also employs a proprietary markup language CopasiML, and conversion/validation tools. The results of each simulation are given either in a time course table, SBML or Copasi Native text format; no plots are shown on a graph. CopasiWeb also provides a simulation service, like RoadRunner.

BioUML [50] is open source integrated Java platform for building virtual cell and virtual physiological human. The web edition provides model visualization, editable model description (both visual and table style), model upload (only specific user types). At the time of our testing, the simulation did not work, throwing “Unable to fetch diagram beans/process/1” error. Visual model editing also gave an error “Element not found” when as a user, we tried to move or modify elements.

The BMOND (Online Database) data is collected from SBML model repository [51] and CellML model repository. It provides a static diagram, which cannot be zoomed. There are many exceptions after clicking a node.

BioUML Workbench [52] is a java-based client application. The program is downloaded which mismatches with the online manual. At the time of testing (February 2012), the program was running, but not functioning well, giving exceptions.

SemanticSBML [53] is a collection of tools for viewing and editing biochemical models in SBML format. SemanticSBML does not have a simulation module, works on Firefox, and has the following functionalities (At the time of testing (February 2012), the system kept giving inconsistency errors while testing functionalities): (i) view: This is a static view (.svg) which is rendered at the time of page load. User cannot move the components on the visualization. (ii) Dynamic manipulation: Users can change the location of the nodes, but cannot edit them, (iii) Browsing: Displays notes, species, and reactions information. PathCase-SB displays this information as well, and also allows the user modify species and reaction information, (iv) Fid Similar: Preloaded models (from BioModels Database) are compared with the model at hand. In comparison, PathCase-SB provides a similarity index in the Model Composition interface, (v) Annotate: Detailed information about Compartments, species, reactions, parameters in the model is shown (similar to PathCase-SB), (vi) Parameters: Parameter balancing can be achieved with SBtab files, (vii) SBtab: validates or converts SBtab files, (viii) SBO terms: Displays SBO Terms, similar to PathCase-SB, (ix) Diff/Merge/Split: Displays the differences between two models. At the time of testing, Merge or Split functions did not work, giving errors. PathCase-SB Model Composition Interface has similar, but more expanded, functionalities, (x) JWS: This functionality provides annotation of jws_online.

## Conclusions

PathCase-SB version 1 is currently available, and being accessed by a small number of users around the world (only for the purposes of statistics gathering, we are monitoring the use of PathCase-SB). We expect that, once we (a) add hierarchical model composition into the Model Composition Interface, and (b) integrate CellML-based models into PathCase-SB, the usefulness and the use of PathCase-SB will increase.

We have many plans to improve and add capabilities to each of the interfaces. The most significant extension is with respect to the Model Composition Interface: we are following the SBML Level 3 hierarchical model composition proposal [37], and the related upcoming LibSBML [29] development effort. Once the proposal is approved and LibSBML has the accompanying features, we plan to provide support for the SBML level 3 hierarchical model compositions in our Model Composition Interface.

Next we list additional queries that rely on model hierarchies, to be implemented after model hierarchies are incorporated into PathCase-SB: (i) List molecular species modeled in a given hierarchy of models (where the hierarchy here may be due to a component hierarchy or complexity-based hierarchies of the same process, etc.), (ii) List model hierarchies of a multi-component model, or of a given set of models, (iii) List models containing a reaction that is n-step downstream/upstream from the molecular species in a model, (iv) List models that contain reactions of a given pathway in a given biological system (cell, tissue/organ), (v) List models that contain a reaction mechanism (e.g. inhibition and/or activation) for an enzymatic reaction of a given pathway, (vi) List models that contain mechanisms (reactant, product, modifier, inhibition and activation) for a reaction, and (vii) List model by category (e.g. gene expression, metabolism, cell cycle, enzymology).

Extensions to the PathCase-SB provenance data include: (a) displaying the provenance data for user-uploaded models, (b) displaying detailed timestamp and RoadRunner simulation service information for uploaded model simulation results (as a process lineage), and (c) querying provenance data on PathCase-SB System as a help for users to search for targeted provenance information.

Finally, creating an iPad application for iPathCase-SB (replacing the current web interface with much more natural touch screen-based tools of iPad) is yet another planned extension. Three such applications are already created [12]: iPathCase-SMDA (released and available in Apple Store), iPathCase-KEGG, and iPathCase-RCMN (to become available in Apple Store in early February 2012).

## Availability and requirements

PathCase-SB is freely available for use at http://nashua.case.edu/PathwaysSB/Web. **Operating Systems:** PathCase-SB is accessed from a browser; therefore, it is platform-independent.

**Browsers:** PathCase-SB is extensively tested with browsers Internet Explorer and FireFox. It is also tested to a lesser degree with Google Chrome and with Safari browsers.

**Other Requirements: (a) Ajax and JavaScript.** PathCase-SB makes use of Ajax, which is a way of sending data between a web server and client asynchronously with normal page requests. This helps PathCase-SB remain quick and responsive even if there are a substantial amount of data to display at once. For this and other features of the site to work (such as collapsing windows), JavaScript must be enabled in the browser. All modern web browsers such as those mentioned above support JavaScript, but it is possible to have personal security settings in place that prevent JavaScript from running. If certain portions of the site do not appear to be working properly, the user must ensure that JavaScript is enabled and that the browser security settings allow scripting elements to run. **(b) Cookies.** In order to view certain portions of the site correctly PathCase-SB occasionally uses cookies, which are small bits of text stored on a local system's hard drive. Users must ensure that cookies are enabled in the browser in order to get the most out of PathCase-SB. **(c) Java Runtime Environment**. The PathCase-SB Graph Viewer uses the Java software platform. In order to view the applet, version 1.6(also known as version 6) or later of the Java Runtime Environment must be installed on the system from which the viewer is accessed. Users should download and install the latest version of the free plug-in, JRE 1.6 (also known as version 6.0). Some browsers may not allow Java applets to run because of security concerns; if the JRE is installed properly and the Graph Viewer still does not appear, the user should make sure that the browser's security settings allow Java applets (or in the case of Internet Explorer, ActiveX controls). **(d) Monitor Resolution**. While PathCase-SB has been designed to gracefully fit into just about any monitor resolution, it is best viewed at resolutions of 1024x768 pixels and up. 800x600 and 640x480 resolutions will work, too, but the results may not be quite as pleasing to the eye. (e) **Server-side implementation.** Server-side implementation of PathCase-SB is done with ASP.NET Framework using the C# language.

**Any restrictions to use:** PathCase-SB is free to use by academics and nonacademics; there are no restrictions.

## Competing interests

The authors declare that they have no competing interests.

## Authors’ contributions

PathCase-SB design was a collaborative task, led by ZMO, GO, NL, RD, and (late) Marco Cabrera. PathCase-SB implementation was done by SAC (Simulation Interface and Provenance Tool), XQ (Graph Viewer and Browser Interface), AC (all interfaces), EC (Querying Interface), RJ (Browser Interface), AEC (Provenance Tool), and LY (KEGG parser). All authors read and approved the final manuscript.
